# The controversy on choroid plexus function in cerebrospinal fluid production in humans: how long could different views be neglected?

**DOI:** 10.3325/cmj.2015.56.306

**Published:** 2015-06

**Authors:** Darko Orešković, Marijan Klarica

**Affiliations:** 1 Ruđer Bošković Institute, Department of Molecular Biology, Zagreb, Croatia doresk@irb.hr; 2 Department of Pharmacology and Croatian Institute for Brain Research, School of Medicine University of Zagreb, Zagreb, Croatia marijan.klarica@mef.hr; * Both authors contributed equally to this article.

The aim of this article is to provide a different perspective to choroid plexus (CP) physiology and pathophysiology from that presented in the recently published review by Spector et al (1). This review with an extensive insight into the relevant literature data had an intention to interpret CP functions focusing on adult humans. The CP is claimed to be the key “organelle” for interpretation and understanding of the classic cerebrospinal fluid (CSF) physiology hypothesis. Namely, it is believed that CSF is actively formed mainly by the CP inside brain ventricles, after which it circulates from the ventricles through the entire CSF system to be passively absorbed into the venous sinuses and/or via paraneural sheaths of nerves into the lymph (2-5). Since CSF secretion by the CP is an active process, it seems logical that CSF secretion is the main generator of CSF circulation if (physiological) volume of CSF is to be maintained within the CSF system. In other words, CSF secretion and absorption inside the CSF space should be balanced, because the amount of secreted CSF must be the same as the amount of passively absorbed CSF. Any other relationship would result in an imbalance of CSF volume, and in time, a pathological process (4). The CP is, in fact, presented as a biological pump by which CSF is produced in an active process, since the CSF formation rate (secretion) should not be significantly altered by moderate changes in the ventricular pressure (3,5).

In this article we would like to present a different perspective from the mainstream view described by Spector et al (1). Throughout the years, as we have been presenting our views, we have never stated that the CP does not produce CSF and that it is not the place of dynamic exchange in CSF (as misinterpreted in the mentioned article), but instead we have criticized the concept of the CP as a CSF pump and the main source of CSF formation.

It is necessary to stress that the basic understanding of the CP relation to CSF formation is still incomplete and speculative, and that the key experiments were very often conducted in *ex vivo* and *in vitro* conditions, which could significantly differ from those in living organisms (4). Furthermore, the experiments on CP usually traced the substance after it had been released from the blood into the CSF, but did not simultaneously trace its movement from CSF into the blood (both directions). Thus, it was difficult to conclude what was the net income of the substance from the blood into CSF. Anyhow, as shown in the above mentioned review (1), numerous experiments in various animal species undoubtedly demonstrated that the CP was the place where many substances from the blood enter the CSF.

Subsequently, our group proposed a new hypothesis of CSF physiology stating that CSF production and absorption (CSF exchange) were constant and present everywhere in the CSF system and partially in the CP, mainly as a consequence of water filtration between the capillaries and interstitial fluid (4,6-8). Because of this different approach to CSF physiology, a considerable part of the recently published review (1) is devoted to the criticism of our experimental results and ideas. Since the main intention of Spector et al (1) was to present physiology of CP relevant to the adult humans, in this essay we intended to show the obtained results mostly on humans, which in addition overlap to those in the experimental animals.

## Entry of water into the CSF system

Although examination of substances passing through CP is important, one must not forget that those substances account to (less than) 1% of total CSF volume, whereas the remaining 99% accounts to water (4). For this reason, in terms of CSF formation volume, the main question is how water enters the CSF system.

However, experiments that studied means of water entry from the blood to CSF failed to recognize the CP as the site of entry. Recently, water flux from blood into the CSF of the third ventricle has been examined by an MRI technique (JJVCPE imaging). A series of highly sophisticated experiments were performed in control and AQP-1 or AQP-4 loss of function mice (9). It was clearly demonstrated that water influx into CSF was regulated by AQP-4 (outside the CP), known to be responsible for water homeostasis of the pericapillary space, and not by AQP-1 found in the CP, ie, there was no significant contribution of CSF by CP because there was no difference between AQP-1 knockout mice and control animals. Spector et al (1) made two objections to those experiments: first, that the relevance of the results in mice remains unknown in humans, and second, since such small amount of CSF is formed in a short time frame of experiments (66 min), the CP participation in CSF formation is invisible in the total CSF volume. However, both objections can be explained by early human studies of Bering (10). The effect of bilateral choroid plexectomy on appearance of water in CSF system has been studied in human patients. In spite of radical surgical removal of both ventricular CPs, no alteration in the water exchange (D_2_O appearance) could be detected before and after choroid plexectomy. The curves of D_2_O could almost be superimposed on each other (10). The author concludes: *“... if the CP were responsible for a major fraction of the water exchange, the postoperative appearance of D_2_O should have been much slower than preoperatively. This is proof positive that CP have at most only a small part in the water exchange of the CSF of the cerebral ventricles.”* (10). It is important to stress that duration of these experiments was 180 minutes and that, according to the classic hypothesis, one would expect at least 36 mL of newly formed CSF, ie, an amount of CSF which should be observed in collected results. Hence, even in longer studies in humans, the results were analogous to those obtained in animals, ie, no significant contribution of the CP to water influx was observed.

Very similar results were also obtained in dogs, where overlapping ^3^H_2_O curves within the lateral brain ventricles and cisterna magna suggested the same mechanism of water entry from the bloodstream into these compartments in terms of dynamics and volume (11). But if the CP is the place of CSF formation, these two curves should differ substantially, with the highest ventricular values at the CP location. Furthermore, very recently new research results have showed oxygen enhancement in ventricular and subarachnoidal (sulcal) CSF (12). These results, obtained on 15 healthy volunteers using spin eco MRI sequence, support the idea that cerebral vessels are involved in CSF production, and reveal that the CSF signal rapidly increases after oxygen administration in both sulcal and ventricular CSF, with significantly more predominant increase in sulcal CSF. These findings also correspond with the results obtained in humans by Bering (10), according to which the appearance of D_2_O was faster in the cisterna magna than in brain ventricles. This fact, together with the faster appearance of oxygen in the sulci, led to an obvious conclusion: appearance of substances (D_2_O and oxygen) cannot be a consequence of CSF flow from the cerebral ventricles. According to all this, it should be concluded that CPs in humans only partially contribute to the CSF/water volume.

Furthermore, it is well known that CSF communicates freely with the brain extracellular fluid (4). There is no universal CSF in terms of fluid composition, since it depends on the site from which it was sampled (2,13). In other words, the biochemical composition of CSF differs depending on the CSF compartment, meaning that exchange of other substances, and not only water, between CSF and surrounding tissue takes place everywhere inside the CSF system. In this case, it is obvious that CP cannot be an exclusive and dominant site of CSF exchange.

## Choroid plexus and hydrocephalus

No one has done so much for the promotion of the idea that the CP is the site of CSF formation as professor Dandy (14), connecting it closely to etiopathogenesis of hydrocephalus. Based on an experiment on a dog (14), he concluded that CP was the exclusive site of CSF formation, that the formation was an active process, and that the blockage between the CP and the site of CSF absorption led to hydrocephalus development in front of the blockage. Such interpretation of hydrocephalus etiology persists to this day, even though it can hardly be applied to numerous clinical observations (8). Consistently to his experiments, Dandy introduced choroid plexectomy as a surgical principle of hydrocephalus treatment. If CP really was the site of CSF formation, choroid plexectomy should stop CSF accumulation and eventually cure hydrocephalus. For many years this was the most popular form of treatment for infantile hydrocephalus in the United States. However, it became clear that bilateral extirpation or cauterization of the choroid plexuses invariably failed to benefit the patients. Because of universally poor results, choroid plexectomy was abandoned by neurosurgeons, and has no place in the current treatment of hydrocephalus. Disadvantages of Dandy’s crucial experiment (14) have been thoroughly analyzed and presented (4), but the failure of choroid plexectomy to cure hydrocephalus is evidence enough that the CPs are not the main source of active CSF formation.

With the development of endoscopic methods in the mid 1990s, new attempts were made to cure hydrocephalus with different surgical procedures on the CP (4,15,16). Although the results were somewhat better than those obtained by the classic surgical approach, the same problems still persisted. The ventricular size was not significantly reduced by CP coagulation, and only 35% of the patients achieved long-term control without cerebrospinal fluid shunts (15). Another study (17) showed that shunting was required in 48% of the cases, which was done from 1 week to 13 months after the CP coagulation. And even when the CP was removed, the development of hydrocephalus still occurred. A recent report showed that it was necessary to perform dual shunting in a male infant with idiopathic CSF overproduction in spite of bilateral ventricular endoscopic CP coagulation, because CSF production was still 700-800 mL/d (16). All this shows that the role of CP in the pathophysiology of hydrocephalus is still unclear and that our knowledge about this process is insufficient. It also clearly confirms the mentioned claims about the CSF formation related to the CP.

## CSF circulation

For some reason, Spector et al (1) described our experimental results on the absence of the CSF flow (circulation) as unexpected. They can hardly be regarded as unexpected since even Bering wrote in 1974 (13): *”The common concept that CSF flows slowly but steadily from the cerebral ventricles into the subarachnoid space up over the cerebral hemispheres to the arachnoid villi is not the case, and it leads to misinterpretation of experimental data and clinical interpretation.”* Furthermore, our findings on the absence of CSF circulation are anything but unexpected, since our group has continuously been publishing articles on CSF “circulation” since 1991 (4). However, our article that Spector et al mention (18) is less relevant to the subject of “circulation” than our articles that they did not mention (4,6-8,19-21). Moreover, Spector et al noted that CSF circulation was shown by an MRI technique, yet omitted to mention the cases in which MRI scans showed the opposite (4). Thus, recently developed Time-Spatial Inversion Pulse (Time-SLIP) method allows direct visualization of the CSF flow using MRI. In this method, the CSF itself serves as an endogenous tracer when radiofrequency pulses are applied (22). Results obtained in humans suggest that there is no unidirectional CSF circulation, and that CSF does not flow from the brain ventricles to the arachnoid villi. Instead, only pulsatile to-and-fro movement of CSF in circadian rhythm is observed.

## Controversy regarding the CP

It is unquestionable that CSF is formed within the ventricular cavities of some lower vertebrates without CPs (4,23,24). It is also well known that it is formed within the neural tube of fetal pigs (25) and humans (23) even before the choroid plexuses anlage appears (26). Therefore, during the entire lifetime in some species, or only during embryonic/fetal development in others, CSF is normally produced although CPs (as the main place of CSF secretion) do not exist. A very similar case has recently been reported in a patient with hydranencephaly and macrocephaly (27). A thirty-year old female presented with an extremely large intracranial space and hydrocephalus mainly filled with CSF (CSF occupied approximately 95% of the cranial cavity) and with only small areas of sustained parenchyma. Since the patient had no CPs inside the supratentorial space and no obstruction between the dural sinuses and CSF, the development of hydrocephalus and macrocephaly could not be explained by the classic hypothesis. Equally difficult to explain were the constant everyday presence and turnover of such a large amount of CSF, as well as maintenance of CSF homeostasis for over 30 years in the absence of CPs.

Furthermore, in spite of the presence of the CPs in isolated brain ventricles in some sharks, there was no open communication between internal and external CSF and no tendency for ventricular dilatation in physiological conditions under stable and constant presence of CSF in subarachnoid space (4,23,24). A similar case has recently been published, which reported on a female patient (28) who presented with a large pineal cyst obstructing the aqueduct of Sylvius, with complete absence of CSF movement through the aqueduct and without development of hydrocephalus over at least 5-year observation period. Additionally, she had no history of clinical symptoms such as headache, nausea, vomiting, ataxia, dementia, etc. In light of the presumption that CPs represent CSF pumps, the absence of hydrocephalus is practically unexplained. Also in this patient it is difficult to explain CSF homeostasis with a constant CSF turnover behind the obstruction site. Namely, there is a considerably larger volume of CSF behind the obstruction than in ventricles (10:1), which should be sustained only by one CP located in IV ventricle.

Spector et al have misinterpreted the article of Lorenzo and Sondgrass (29) by concluding that perfusion experiments in cats provided unequivocal evidence that CSF was formed in ventricles exclusively, but not inside the subarachnoid space. We have thoroughly discussed and explained that perfusion experiments were not a reliable method for calculation of CSF formation (30). Regardless of that, since both ventriculo-cisternal (cistern magna; CM) and ventriculo-subarachnoid (cortex) perfusion occupy a significant part of the subarachnoid space, based on the comparison of both perfusion results it cannot be concluded that CSF is formed exclusively inside the ventricles. CSF inside the CM does not originate solely from the ventricles, but also from basal cisterns and the spinal space. For this reason, the surface of CSF system included in both perfusion methods is not so different, and 14% higher rate of CSF formation (although not statistically significant) observed during ventriculo-cortical perfusion (29) also fails to speak in favor of ventricular CSF formation hypothesis.

Although the above-mentioned results and controversies do not fit into the classic hypothesis, they can be explained by the new hypothesis of CSF physiology, which offers a different perspective on CSF physiology and pathophysiology (4,8,11,31,32).

The fact that after plexectomy the patient continues living with normal CSF turnover (4,15,33,34) indicates that the CP is not of vital significance for a living organism. It is a highly vascularized structure immerged into the brain ventricles' CSF, with an expressed active metabolic nature (1,4,35). Based on all of the mentioned above, one could presume that (in terms of evolution) getting the blood vessels in close contact with the CSF volume inside of brain ventricles could accomplish a significant and fast matter exchange between the blood and CSF, and vice versa, with a purpose of maintaining the biochemical balance of CSF as an important physiological medium for the normal CNS functioning.

## Conclusions

The controversy regarding the role of brain plexuses is not yet at the point to be resolved. The cumulating evidence and application of new technologies, such as MRI, have already made their contributions, and we should further strive to improve our concepts and create the right therapies for patients. Our contribution to this discussion is rather straightforward – the role of CP in the physiology and pathophysiology of CSF shown in the textbooks, atlases, and review articles is highly overemphasized and needs to be revised.
